# Single-Cell Optogenetic Control of Calcium Signaling with a High-Density Micro-LED Array

**DOI:** 10.1016/j.isci.2019.10.024

**Published:** 2019-10-19

**Authors:** Dacheng Mao, Ningwei Li, Zheshun Xiong, Yubing Sun, Guangyu Xu

**Affiliations:** 1 Department of Electrical and Computer Engineering, University of Massachusetts, Amherst, MA 01003, USA; 2 Department of Mechanical and Industrial Engineering, University of Massachusetts, Amherst, MA 01003, USA

**Keywords:** Techniques in Genetics, Cellular Neuroscience, Techniques in Neuroscience, Bioelectronics, Electronic Materials

## Abstract

Precise optogenetic control, ideally down to single cells in dense cell populations, is essential in understanding the heterogeneity of cell networks. Devices with such capability, if built in a chip scale, will advance optogenetic studies at cellular levels in a variety of experimental settings. Here we demonstrate optogenetic control of intracellular Ca^2+^ dynamics at the single cell level using a 16-μm pitched micro-light emitting diode (LED) array that features high brightness, small spot size, fast response, and low voltage operation. Individual LED pixels are able to reliably trigger intracellular Ca^2+^ transients, confirmed by fluorescence microscopy and control experiments and cross-checked by two genetically coded Ca^2+^ indicators. Importantly, our array can optogenetically address individual cells that are sub-10 μm apart in densely packed cell populations. These results suggest the possible use of the micro-LED array toward a lab-on-a-chip for single-cell optogenetics, which may allow for pharmaceutical screening and fundamental studies on a variety of cell networks.

## Highlights

• Precise optogenetic control of Ca^2+^ signaling down to the single cell level
• Bright, localized optogenetic stimulus with a high-density micro-LED array
• Advancing micro-LED arrays toward a lab-on-a-chip for single-cell optogenetics

## Graphical Abstract

**Figure:**
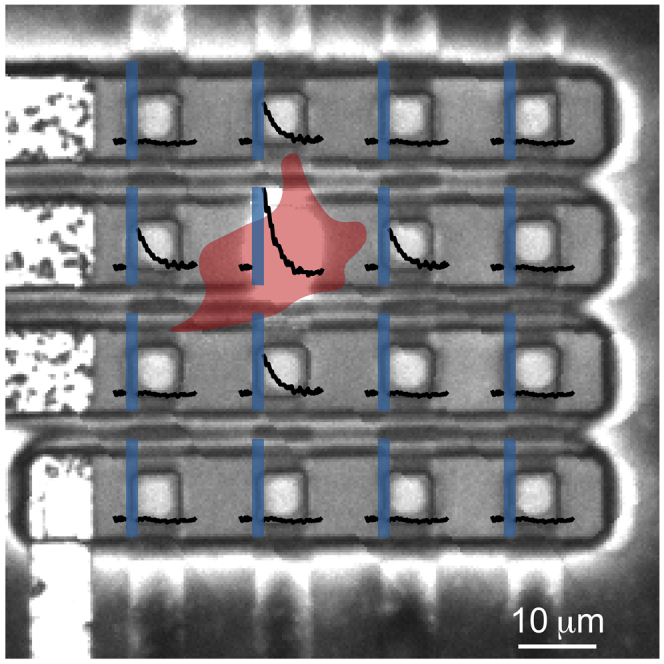

## Introduction

Over the past decade, optogenetics has emerged as a powerful method to interrogate specific cell types in complex tissues (Boyden, 2015, Deisseroth, 2015). In this method, cells expressed with light-sensitive proteins can be *either* excited *or* silenced, when exposed to light pulses at specific wavelengths (Boyden et al., 2005, Prakash et al., 2012). Such bidirectional optical control over the activity of cells enables minimally invasive assessment of their roles at the levels of cells, circuits, and behavior. For these reasons, optogenetics is widely used to modulate the activity of neurons (Chow et al., 2010, Hochbaum et al., 2014), cardiomyocytes (Wang et al., 2017, Johnston et al., 2017), C2C12 myotubes (Asano et al., 2015, Sebille et al., 2017), and human embryonic kidney 293 cells (i.e., HEK 293) (Baaske et al., 2018, Miyazaki et al., 2019), which deepens the understanding of brain/heart/muscle functions and gene expression.

To make the full impact of optogenetics, research has now focused on advancing the hardware to enable precise optogenetic control (McGovern et al., 2010, Nakajima et al., 2012, Steude et al., 2016), ideally down to single cells in dense cell populations. Such single-cell precision is essential in understanding the heterogeneity of cell networks, which is challenging to achieve by electrical stimulation methods using micro/nanoelectrodes (Hierlemann et al., 2011, Abbott et al., 2017, Lu et al., 2018). If successful, the resulting hardware will help scientists find precise connections within and between different tissue regions at an unprecedented cellular level. However, it is technically difficult to express optogenetic actuators in targeted single cells; in fact, densely packed cells often express them simultaneously, thereby all becoming light sensitive. Thus, to achieve single-cell optogenetic control one should employ high-density light sources that can address individual cells by localized light output.

Among available light sources, micron-sized light-emitting diode arrays (i.e., micro-light emitting diode [LED] arrays) are suitable for high-precision optogenetic control (Steude et al., 2016, Poher et al., 2008, Grossman et al., 2010, McGovern et al., 2010, Nakajima et al., 2012, Pisanello et al., 2016). These devices are recognized for their scalability, good lifetime in biological environments, and medium power dissipation for *in vivo* use. To date, GaN-based micro-LED arrays with 50- to 200-μm pitches have been built into the microscope optics for multi-site light illumination at a variety of settings, using a patch clamp or microelectrode arrays to record optogenetically induced action potentials (Grossman et al., 2010, Nakajima et al., 2012). Recently, organic micro-LED arrays with sub-10-μm pitches have been applied to HEK 293 cell culture, employing a patch clamp to monitor the photocurrent in single cells that were illuminated by three LED pixels (Steude et al., 2016). Although these studies showcased single-cell optogenetics using high-density micro-LEDs, studying cell activity in the electrical domain only is insufficient to fully understand cellular network dynamics. In fact, cell circuits often not only involve the transmission of electrical signals among cells but also associate with complex synaptic chemistry (Garris, 2010, Andrews, 2013). These chemistries correlate with each other, play key roles in regulating cell activity, and add to high-content analysis of cell signaling. For instance, intracellular calcium concentration (i.e., [Ca^2+^]) is an essential biochemical signal in the regulation of muscle contraction, neurotransmitter release, and gene expression (Berridge et al., 2003, Clapham, 2007, Tu et al., 2016, Grewe et al., 2010, Seta et al., 2004).

To this end, here we demonstrate precise optogenetic control of intracellular Ca^2+^ dynamics at the single cell level using a 100%-yield, 16-μm pitched micro-LED array that can output bright, localized, and fast-switching light in low-voltage operation. Single LED pixels are able to reliably trigger intracellular Ca^2+^ transients, evidenced by fluorescence microscopy, control groups, and comparative studies using two complementary Ca^2+^ indicators. Importantly, our array can optogenetically address individual cells that are sub-10 μm apart in densely packed cell populations. Our results suggest the promise of the high-density micro-LED array toward a lab-on-a-chip for single-cell optogenetics. Combined with its highly scalable structure, this device may enable exciting opportunities in pharmaceutical screening and cell signaling studies in a variety of cell networks.

## Results

**Figure 1:**
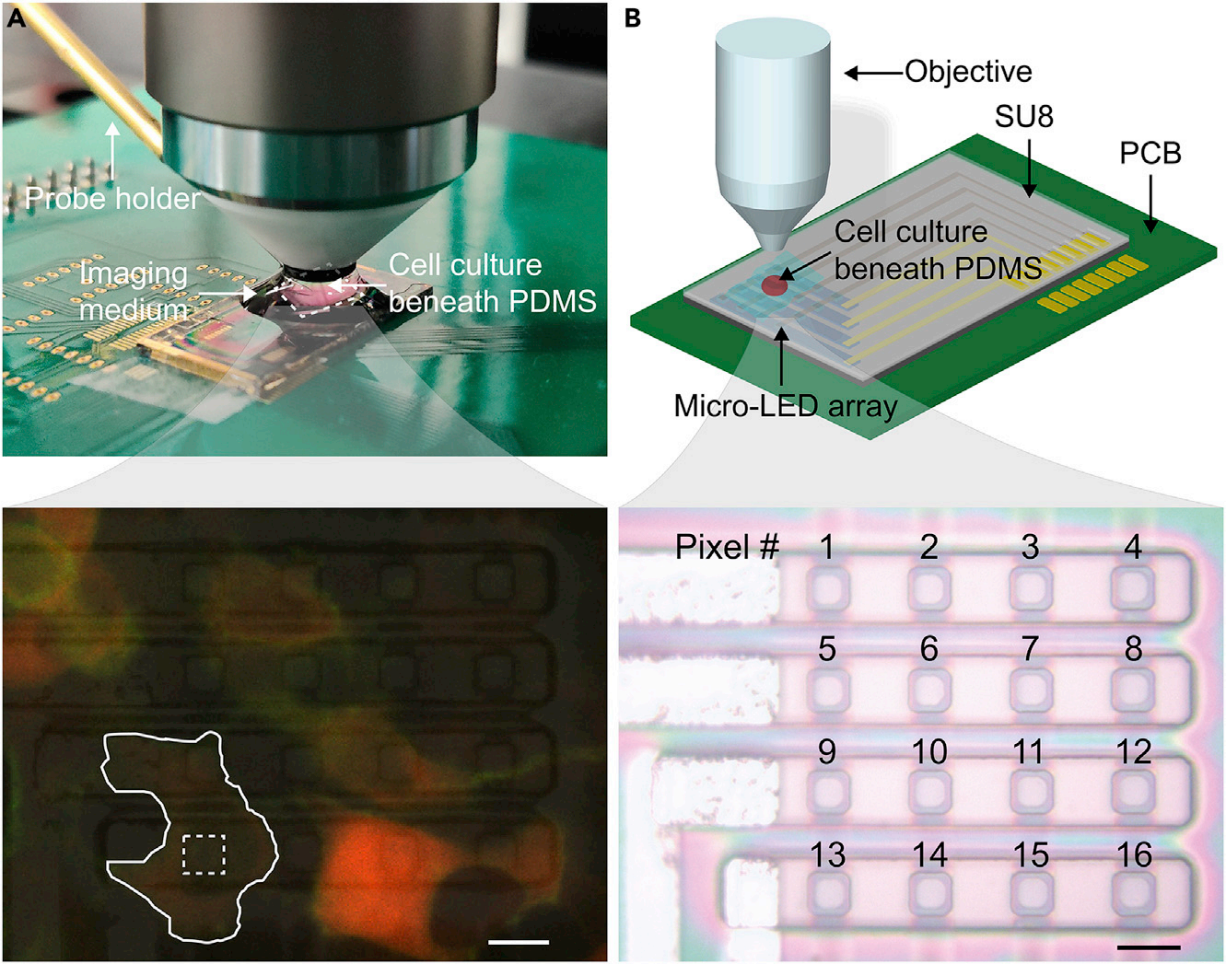
Experimental Setup (A) One micro-LED array wired bonded onto a printed circuit board (PCB) under an upright fluorescence microscope configured for cell imaging. A flipped PDMS piece, seeded with cells on its top surface, was aligned to LED pixels by a probe holder; this way *ChR2* (green)-*jRCaMP1a* (red) co-expressed cell (outlined) spatially overlapped with the LED pixel (squared). Scale bar, 10 μm. (B) Illustration of the experimental setup using a 4-by-4 micro-LED array. Scale bar, 10 μm. See also Figures S1–S4.

To conduct Ca^2+^ imaging under optogenetic stimulus, the activation spectrum of the optogenetic actuator and the excitation spectrum of the Ca^2+^ indicator need to be well separated from each other. This way we can minimize the optical cross talk between the excitation light (used for Ca^2+^ imaging) and the activation light (used for optogenetic control). Therefore, we selected two genetically coded Ca^2+^ indicators, *jRCaMP1a* and *NIR-GECO1* (Dana et al., 2016, Qian et al., 2019), each paired with an optogenetic actuator, *ChR2* (Zhang et al., 2007), and co-expressed them (*either ChR2* with *jRCaMP1a or ChR2* with *NIR-GECO1*) in HEK 293 cells seeded on a polydimethylsiloxane (PDMS) piece (Figures 1A and S1). We note that *NIR-GECO1* is an inverse response indicator to [Ca^2+^] change, which is opposite to *jRCaMP1a*. Therefore, a comparative study using these two complementary Ca^2+^ indicators can cross-check the effectiveness of the optogenetic stimulus applied in the cell experiment.

On the hardware side, we fabricated a GaN-based, 4-by-4 micro-LED array that can output 462/19 nm light to activate *ChR2* (Figure S2). This device was built on commercial epitaxial GaN-on-Si wafers, formed by sequentially growing multiple GaN-based layers on top of a (111) Si substrate (see Figure S3 and Transparent Methods). Using reactive-ion etching steps, a total of 16 LED pixels, each 6.5 μm-by-6.5 μm in size, were patterned in a cross-bar structure with a 16-μm pitch (Figure 1B). We noted that this pitch size is three times smaller than previous GaN-based LED arrays used for optogenetics (Poher et al., 2008, Grossman et al., 2010, McGovern et al., 2010) and close to the typical diameter of a HEK 293 cell. The column and row select lines of the array were formed by Ni/indium tin oxide (5/120 nm for p-GaN contacts) and Ti/Al/Ti/Au layers (10/70/10/120 nm for n-GaN contacts), respectively, and passivated by plasma-enhanced chemical vapor deposition-based SiO_2_ (PECVD- SiO_2_) layers. The array was then encapsulated by another PECVD-SiO_2_ layer (∼200 nm) with a cross-linked SU8 layer on top and wire-bonded onto a printed circuit board for pixel selection (Figure S4). During cell experiments, we flipped a PDMS piece seeded with cells and placed it onto the encapsulated array (Figure 1B). This way, cells faced the LED pixels and could be aligned to the pixel of interest by moving the PDMS piece with a micro-manipulator (see the probe holder in Figure 1A).

**Figure 2:**
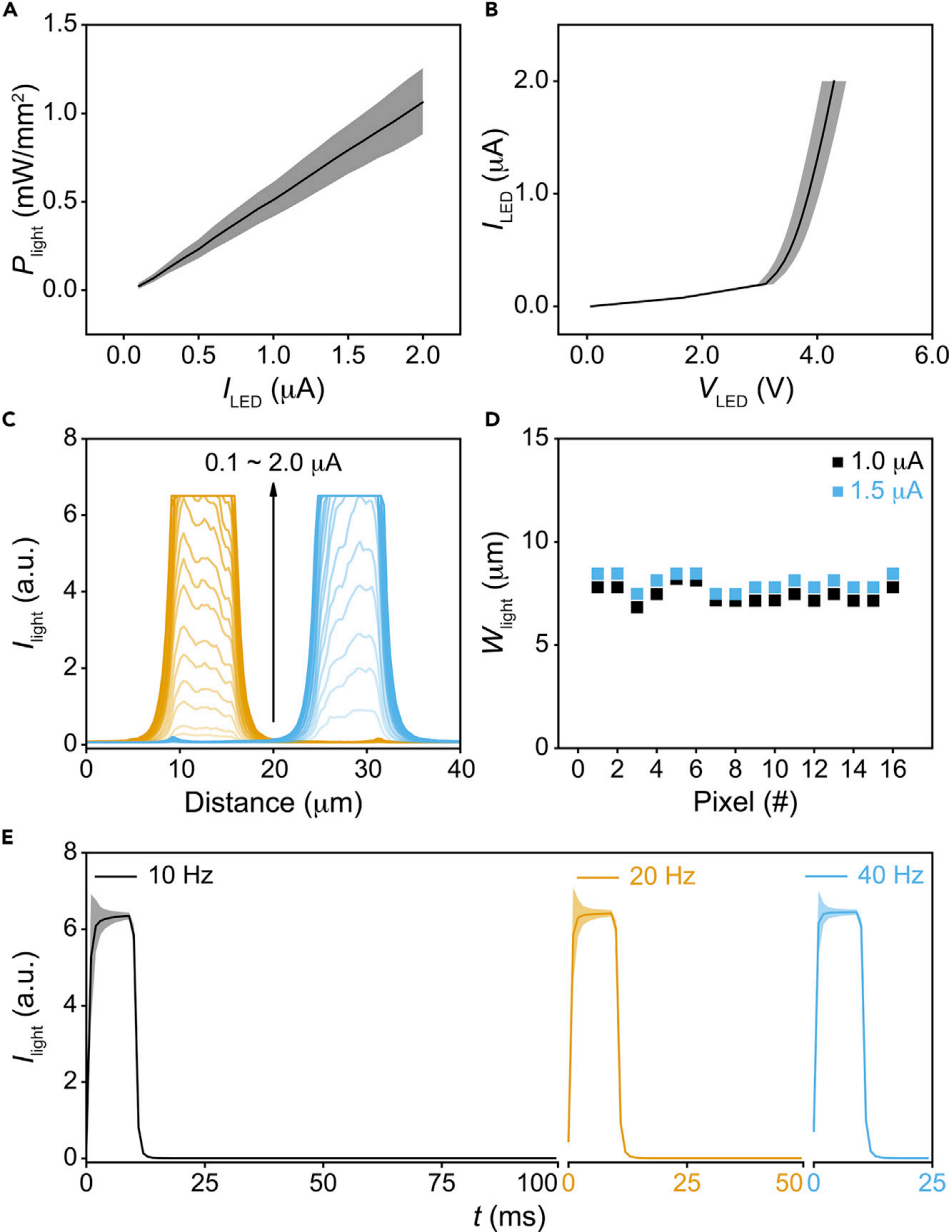
Array Characterization (A) *P*_light_ versus *I*_LED_ for all 16 pixels. (B) *I*_LED_-*V*_LED_ curves for all 16 pixels. (C) Spatial profile of the pixel output (from two neighboring pixels) at the array surface with *I*_LED_ ranging from 0.1 to 2.0 μA. (D) FWHM values of all 16 pixels with *I*_LED_ = 1.0 and 1.5 μA. (E) Pixel output pulsed with a 10-ms pulse duration at 10- to 40-Hz pulsing frequencies. The pixel was biased at *I*_LED_ = 2.0 μA. In (A), (B), and (E), shaded areas represent ±1 SD.

To enable optogenetic control at the single cell level, the micro-LED array is required to output bright, localized, and fast-switching light, ideally in a low-voltage operation. To this end, we first measured the optical power density (*P*_light_) and the spatial profile of the illumination spot (*I*_light_) of each LED pixel, using an optical power meter and a fluorescence microscope, respectively. When biased at injection currents (*I*_LED_) ranging from 0.1 to 2.0 μA, all 16 pixels show high brightness with *P*_light_ ∼ 0.1–1.0 mW/mm^2^ (Figure 2A), which falls into the range required for optogenetic control in HEK 293 cells and mammalian neurons (Boyden et al., 2005, Steude et al., 2016, Morton et al., 2019). We note that this high brightness is achieved with the driving voltage across each pixel (*V*_LED_) being less than 4.8 V (Figure 2B). This low-voltage operation leads to sub-10-μW electrical power dissipation per pixel, which may ultimately allow for *in vivo* use (Marblestone et al., 2013, Mao et al., 2018). Moreover, at *I*_LED_ = 1.0 or 1.5 μA, all 16 pixels output small light spots with the full width at half maximum (FWHM) < 10 μm at the array surface (Figures 2C and 2D, the FWHM is overestimated here as the center of the light spot in some pixels saturates the camera). Importantly, this localized pixel output with *P*_light_ ∼ 0.5–0.8 mW/mm^2^ is encouraging for optogenetic control over single cells close to the array surface, as HEK 293 cells are ∼10 μm in size. Finally, we found that such bright, localized pixel output (i.e., *P*_light_ ∼ 0.5 mW/mm^2^, FWHM <10 μm) can be pulsed with a 10-ms duration at up to 40-Hz pulsing frequencies (Figure 2E). After 3 ms in each pulse, the pulsed light intensity approached to its steady state value, with <3% variation among all pulses during 1-s recording. These results suggest that our LEDs meet the brightness, resolution, and speed requirement for optogenetic studies at cellular levels.

To prepare the optogenetic experiments, we flipped the PDMS piece—seeded with HEK 293 cells on its top surface—and placed it onto the array to let cells face the LED pixels. This PDMS-flipping approach adds to single-cell optogenetics in two ways. First, by getting cells closer to the array, this approach enhances the amount of light each cell receives and thus the strength of optogenetic control. Second, since LEDs emit light omnidirectionally, the spatial resolution they can offer is better close to the array surface, where the light spot is smaller. On the biology side, we cultured HEK 293 cells on sterilized PDMS pieces at 37°C in a humidified incubator, added the co-factor *all-trans retinal* to enhance light transduction of *ChR2* (Steude et al., 2016, Cho et al., 2019), and transfected one of the two Ca^2+^ indicators with (co-transfection) or without (single transfection) *ChR2* into these cells, all following the manufacturers' recommended protocols (see Transparent Methods). Before optogenetic experiments, we added an imaging solution containing 80 mM CaCl_2_ to cell culture, which serves to enhance cell responses to optogenetic stimulus as more extracellular Ca^2+^ would flush into the cell when *ChR2* gets activated (Cho et al., 2019, Lin et al., 2009).

With these preparation steps, we next conducted optogenetic experiments using a standard Ca^2+^ imaging configuration by a fluorescence microscope. During each experiment, we pulsed 575/25-nm excitation light using the microscope with 0.5 frame per second and 100-ms exposure time per frame to alleviate the photo-bleaching effect. Meanwhile, the cell of interest was optogenetically stimulated by LED pixels in three consecutive recording periods. In each period, we illuminated the select LED pixel 20 s after the Ca^2+^ signal of the cell reached the steady state, with the excitation light being shut off at the same time. Here we chose not to collect Ca^2+^ imaging data during the optogenetic stimulation since the LED light would otherwise leak through the emission filter, introduce an artifact in the Ca^2+^ imaging data, and obscure the analysis. In parallel, we also conducted experiments with the optogenetic stimulus being provided by the microscope (3.92 mW at 470/24 nm), in which case all cells in the field of view were illuminated simultaneously.

**Figure 3:**
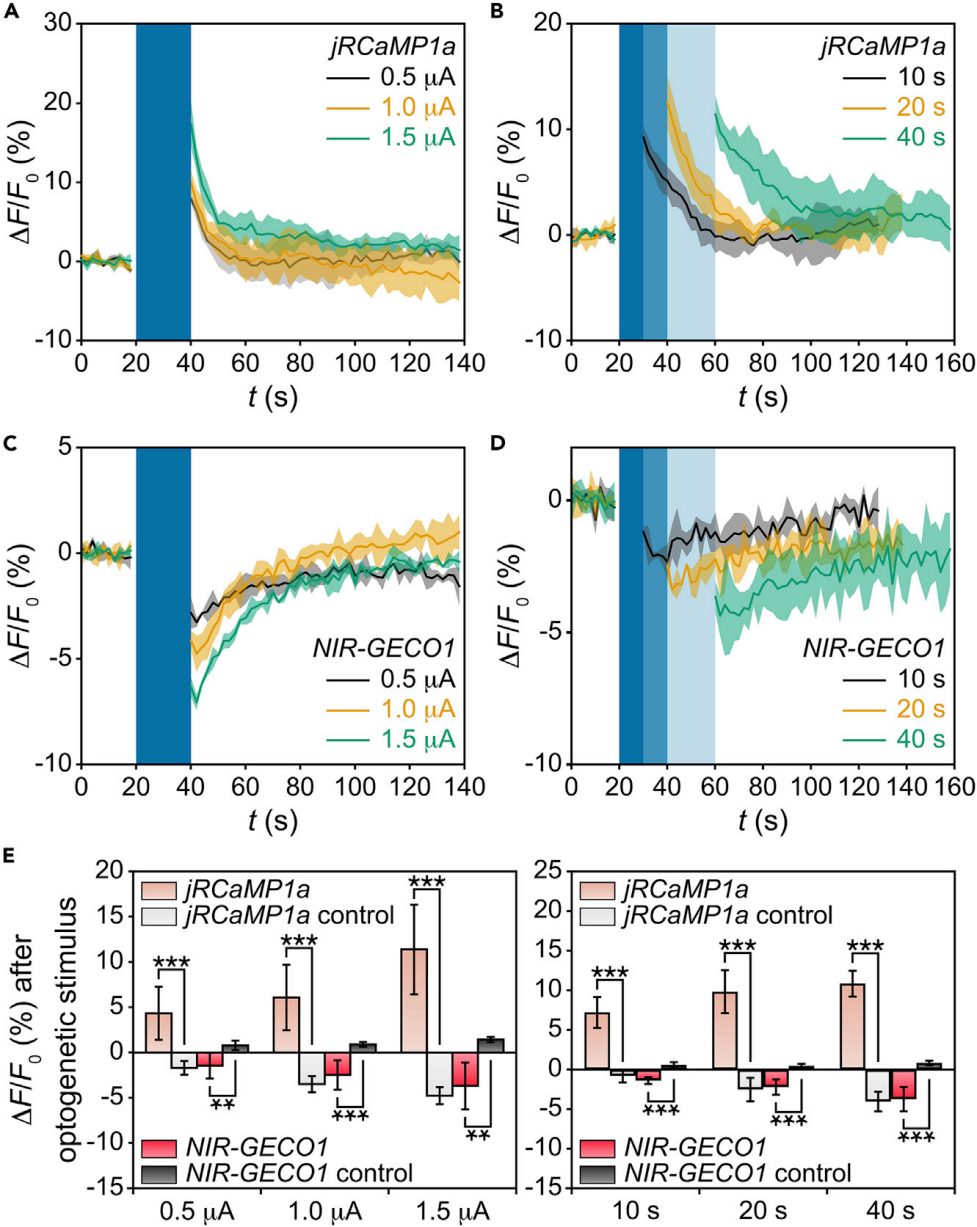
Cell Responses to Optogenetic Stimulus Offered by LEDs (A) *ΔF/F*_*0*_ traces from a *ChR2-jRCaMP1a* co-expressed cell with *T*_LED_ = 20 s and *I*_LED_ ranging from 0.5 to 1.5 μA. (B) *ΔF/F*_*0*_ traces from a *ChR2-jRCaMP1a* co-expressed cell with *T*_LED_ ranging from 10 to 40 s and *I*_LED_ = 1.5 μA. (C) *ΔF/F*_*0*_ traces from a *ChR2-NIR-GECO1* co-expressed cell with *T*_LED_ = 20 s and *I*_LED_ ranging from 0.5 to 1.5 μA. (D) Representative *ΔF/F*_*0*_ traces from a *ChR2-NIR-GECO1* co-expressed cell with *T*_LED_ ranging from 10 to 40 s and *I*_LED_ = 1.5 μA. In (A), (B), (C), and (D), blue windows represent the periods of optogenetic stimulus; solid lines represent the mean values from three consecutive recording periods; shaded areas represent ±1 SD. (E) *ΔF/F*_*0*_ signals versus *I*_LED_ (left) and *T*_LED_ (right) in both co-expressed and control cells. Error bars represent ±1 SD (*n* = 9 from three independent cells in each group, three recording periods from each cell); **p < 0.01, ***p < 0.001 based on Student's t test. See also Figures S5 and S6.

After each optogenetic stimulation with *I*_LED_ ranging from 0.5 to 1.5 μA and the duration (*T*_LED_) ranging from 10 to 40 s, *ChR2*-*jRCaMP1a* co-expressed cells were found to reliably increase their emitted fluorescence intensities (Figures 3A and 3B). Here we define the *F*_0_ value as the 20-s average before each stimulation, subtracted by the background measured at the dark region in the field of view. The resulting positive Δ*F*/*F*_0_ values after each stimulation suggest an increase of intracellular Ca^2+^ level, coming from the optogenetically triggered Ca^2+^ influx to the illuminated cell (Dana et al., 2016, Morton et al., 2019). To examine if such Ca^2+^ increase was specific to the optogenetic activation of *ChR2*, we conducted control experiments with cells that are transfected with *jRCaMP1a* only (i.e., control cells). Indeed, these cells did not increase their Δ*F*/*F*_0_ values after optogenetic stimulus since no *ChR2* were expressed to assist the Ca^2+^ influx (Figure S5). We confirmed these results by additional experiments with cells being optogenetically stimulated by the microscope (i.e., microscope-based stimulus), which yielded qualitatively similar Δ*F*/*F*_0_ traces in both co-expressed and control cells (Figure S6).

On the other hand, we found that *ChR2*-*NIR-GECO1* co-expressed cells reliably decreased their emitted fluorescence intensities by optogenetic stimulus (Figures 3C and 3D). The resulting negative Δ*F*/*F*_0_ values are opposite to that in *ChR2*-*jRCaMP1a* co-expressed cells, because *NIR-GECO1* is an inverse response indicator to the optogenetically triggered Ca^2+^ influx (Qian et al., 2019). In the control experiments, cells only transfected with *NIR-GECO1* did not decrease Δ*F*/*F*_0_ values after optogenetic stimulus since no *ChR2* were expressed to assist the Ca^2+^ influx (Figure S5). Likewise, we confirmed these results by additional experiments with microscope-based optogenetic stimulus, which yielded similar Δ*F*/*F*_0_ traces (Figure S6). These data with *NIR-GECO1* being the Ca^2+^ indicator further validate the effectiveness of the optogenetic stimulus offered by LEDs.

To quantify the strength of the applied optogenetic stimulus, here we define the Δ*F*/*F*_0_ value right after each optogenetic stimulus as our signal. To perform statistical analysis, we collected data from three independent *ChR2*-*jRCaMP1a* co-expressed cells*, ChR2*-*NIR-GECO1* co-expressed cells, or their corresponding control cells. Since each cell was tested in three consecutive recording periods, our statistics is based on n = 9 such periods from three independent cells (Figure 3E).

In *ChR2*-*jRCaMP1a* co-expressed cells, the Δ*F*/*F*_0_ value after optogenetic stimulus is positive and mildly increases with *I*_LED_ and *T*_LED_. This dependence is likely because more LED stimulus, by increasing either *I*_LED_ or *T*_LED_, would increase the amount of Ca^2+^ influx by opening more *ChR2*-related Ca^2+^ channels. In contrast, among *jRCaMP1a* control cells, the Δ*F*/*F*_0_ value after optogenetic stimulus becomes negative (confirmed by additional experiments using microscope-based stimulus, see Figure S6) and mildly increases its amplitude (i.e., absolute value) with *I*_LED_ and *T*_LED_. This negative Δ*F*/*F*_0_ value in *jRCaMP1a* control cells is likely due to the temporary photobleaching of *jRCaMP1a* by the 10- to 40-s constant LED illumination, which was later recovered at the end of each recording period. Another possible reason is that such constant LED illumination may temporarily increase the local temperature (Marblestone et al., 2013) and thus decrease the local pH next to the cell of interest. This local pH decrease can temporarily lower the fluorescence intensity of *jRCaMP1a* as reported before (Oliver et al., 2000, Kerruth et al., 2019, Zhao et al., 2011).

On the other hand, in *ChR2*-*NIR-GECO1* co-expressed cells, the Δ*F*/*F*_0_ value after optogenetic stimulus is negative and mildly increases its amplitude (i.e., absolute value) with *I*_LED_ and *T*_LED_. Again, this dependence is likely because the Ca^2+^ influx increases with the strength of the optogenetic stimulus offered by LEDs. In contrast, among *NIR-GECO1* control cells, the Δ*F*/*F*_0_ value after optogenetic stimulus is found to be positive (confirmed by additional experiments using microscope-based stimulus, see Figure S6) and slightly increases with *I*_LED_ and *T*_LED_. This positive Δ*F*/*F*_0_ value in *NIR-GECO1* control cells is also likely because the 10- to 40-s constant LED illumination temporarily increased the local temperature and thus decreased the local pH next to the cell of interest. Such pH decrease (∼7.3 in the imaging solution) can temporarily enhance the fluorescence intensity of *NIR-GECO1* as reported before (Qian et al., 2019). Another possibility is that such constant LED illumination may get *NIR-GECO1* photoisomerized to a metastable brighter state, which was later overwhelmed by the photobleaching effect at the end of each recording period.

In comparison, we found that *ChR2*-*jRCaMP1a* co-expressed cells were overall brighter and had larger Δ*F*/*F*_0_ signals. *ChR2*-*NIR-GECO1* co-expressed cells were overall inferior in these two aspects, but provided robust reverse response to Ca^2+^ changes, and helped cross-check if the optogenetic stimulus offered by LEDs was effective. In addition, our LEDs can typically generate Δ*F*/*F*_0_ values that are on par with—if not larger than—those generated by microscope-based stimulus (Figure S6). This fact re-affirms that our LEDs can indeed provide reliable optogenetic control of Ca^2+^ signaling.

**Figure 4:**
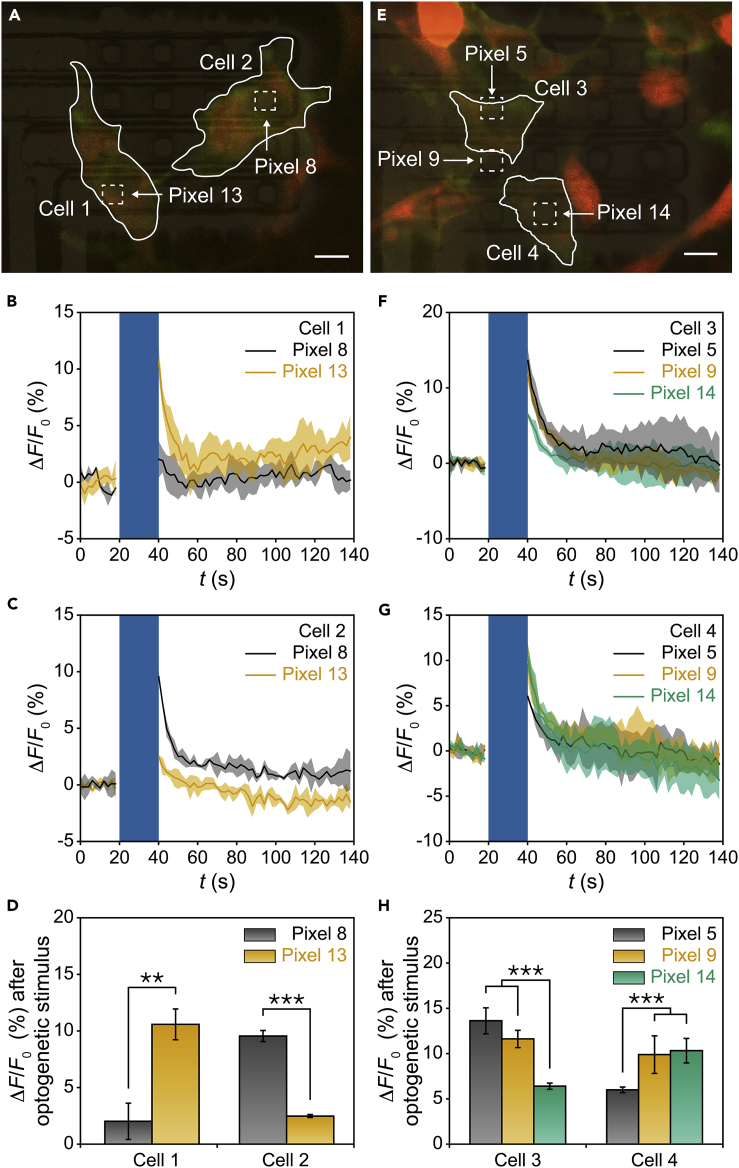
Spatial Resolution of the Optogenetic Stimulus Offered by LEDs (A) One pair of cells (outlined, overlapped with pixels 13 and 8) that were sub-10 μm apart. Scale bar, 10 μm. (B) *ΔF/F*_*0*_ traces of cell 1 stimulated by pixels 8 and 13 with *T*_LED_ = 20 s. (C) *ΔF/F*_*0*_ traces of cell 2 stimulated by pixels 8 and 13 with *T*_LED_ = 20 s. (D) Statistical analysis of *ΔF/F*_*0*_ signals from cell 1 and cell 2. (E) Another pair of cells (outlined, overlapped with pixels 5, 9, and 14) that were sub-5 μm apart. Scale bar, 10 μm. (F) *ΔF/F*_*0*_ traces of cell 3 stimulated by pixels 5, 9, and 14 with *T*_LED_ = 20 s. (G) *ΔF/F*_*0*_ traces of cell 4 stimulated by pixels 5, 9, and 14 with *T*_LED_ = 20 s. (H) Statistical analysis of *ΔF/F*_*0*_ signals from cell 3 and cell 4. In (B), (C), (F), and (G), blue windows represent the periods of optogenetic stimulus; solid lines represent the mean values from three consecutive recording periods; shaded areas represent ±1 SD. The *I*_LED_ values applied to output *P*_light_ ~ 0.71 mW/mm^2^ were 1.2 μA for pixel 5, 1.6 μA for pixel 8, 1.5 μA for pixel 9, 1.0 μA for pixel 13, and 1.1 μA for pixel 14. In (D) and (H), error bars represent ±1 SD (*n* = 3 recording periods); **p < 0.01, ***p < 0.001 based on Student's t test. See also Figure S7.

After validating the performance of our micro-LEDs, we now examine if they can provide precise optogenetic control at the single cell level in dense cell populations. To achieve this, we chose to optogenetically stimulate one pair of neighboring *ChR2*-*jRCaMP1a* co-expressed cells using different LED pixels (Figure 4), all with *T*_LED_ = 20 s and *P*_light_ ∼ 0.71 mW/mm^2^ to compare their evoked Δ*F*/*F*_0_ signals (note: different pixels were biased at different *I*_LED_ owing to pixel-to-pixel variation).

Specifically, cell 1 (overlapped with pixel 13) and cell 2 (overlapped with pixel 8) were sub-10 μm apart with an ∼50 μm center-to-center distance (Figure 4A). Our data in Figures 4B–4D show that (1) cell 1 had significantly larger Δ*F*/*F*_0_ signals when it got stimulated by pixel 13 than when it got stimulated by pixel 8 and (2) cell 2 had significantly larger Δ*F*/*F*_0_ signals when it got stimulated by pixels 8 than when it got stimulated by pixel 13. These results show that *ChR2*-expressed cells indeed responded more to LED pixels that were overlapped with them. Moreover, *either* pixel 8 *or* pixel 13 introduced low cross talk in the cell that was *not* overlapped with the pixel; the Δ*F*/*F*_0_ signal in the cell that was overlapped with the pixel was more than three times that in the cell that was *not* overlapped with the pixel (i.e., selectivity >3). Importantly, these data suggest that our array can indeed address individual cells that are sub-10 μm apart with low cross talk; pixels 13 and 8 can provide such precise optogenetic control over cell 1 and cell 2, respectively.

To examine the limit of spatial resolution our array can achieve, we conducted another experiment (Figure 4E) with two cells even closer to each other. Specifically, cell 3 (overlapped with pixels 5 and 9) and cell 4 (overlapped with pixel 14, spatially closer to pixel 9 than pixel 5) were sub-5 μm apart with an ∼30-μm center-to-center distance. Our data in Figures 4F–4H show that (1) cell 3 had significantly larger Δ*F*/*F*_0_ signals when it got stimulated by pixels 5 and 9 than when it got stimulated by pixel 14 and (2) cell 4 had significantly larger Δ*F*/*F*_0_ signals when it got stimulated by pixels 14 and 9 than when it got stimulated by pixel 5. These results reaffirm that *ChR2*-expressed cells responded more to LED pixels that were overlapped with or closer to them. The fact that cell 4 responded similarly to pixels 9 and 14 is likely because cell 4 was far away from the array surface, where the pixel 9 output was less confined (i.e., spot size increased to >10 μm). However, it is noted that the selectivity in this experiment was less than 3, suggesting that our array cannot address individual cells that are sub-5 μm apart with low cross talk. Taking one step further, we observed that the selectivity was even lower (consistently <2) when cells were sub-1 μm apart (see additional two experiments in Figure S7). We thus conclude that our array can currently achieve sub-10-μm resolution.

## Discussion

In sum, we demonstrated optogenetic control of Ca^2+^ signaling at the single-cell level using a 100%-yield high-density micro-LED array. Our array was found to output bright, localized, and fast-switching light in a low-voltage operation, which can precisely address individual HEK 293 cells that were sub-10 μm apart. Importantly, our results were confirmed by epifluorescence microscopy, control experiments, and cross-checked by two complementary Ca^2+^ indicators, all of which showed statistical significance. This work suggests the promise of the high-density micro-LED array toward a lab-on-a-chip for single-cell optogenetics, which can add to high-content cell signaling studies. Combined with its highly scalable structure, this device may provide a cost-effective platform for pharmaceutical screening and fundamental studies on a variety of cell networks. Leveraging standard semiconductor fabrication steps, our LED arrays can, for instance, readily extend to a medium number of pixels (∼100) to study computational algorithms of the neural network at the *in vitro* setting. On the other hand, we can create alternative versions of the array to output different wavelengths in the visible spectrum. For example, AlGaInP-based micro-LED arrays can be similarly built to output 600–630 nm light, which can be applied to actuate red-shifted opsins (e.g., *Chrimson*) and monitor intracellular Ca^2+^ dynamics using green Ca^2+^ indicators (e.g., *GCaMP7*) at the same time (Klapoetke et al., 2014, Dana et al., 2019).

Finally, we remark that our high-performance array can be used to study single-cell optogenetics in other cell types (e.g., neurons or cardiomyocytes) and provide precise optogenetic control over other cellular signals (e.g., intracellular potassium concentration). For *ex-vivo* or *in vivo* applications our arrays will need to be encapsulated by biocompatible and transparent films (e.g., SU8 or epoxy). Furthermore, if built along a solid-state shank similar to that of the implantable silicon microelectrode arrays (Scholvin et al., 2016), the resulting device would be typically sub-100 μm wide, sub-100 μm thick, and 3–5 mm long, which may ultimately enable single-cell optogenetics in deep tissues. By sequentially illuminating individual pixels, such device would routinely consume sub-10 mW electrical power, which is suitable for long term *in vivo* use. If successful, for instance, one may implant such devices to trigger intracellular [Ca^2+^] change during muscle recovery from injury (van Bremen et al., 2017) or to offer precise modulation of the neurocircuitry in deep brain (Yawo et al., 2013).

### Limitations of the Study

The energy conversion efficiency of our LED pixels, defined as the value of *P*_light_/(*I*_LED_·*V*_LED_) here, suffered from voltage drop across the contact wires. To solve this issue, our array layout will need to be further optimized to reduce the series resistance from these contact wires. On the other hand, we may be able to change the constant LED illuminations to pulsed LED illuminations. We expect such change would alleviate the local heating effect to the cell of interest. Furthermore, by alternatingly pulsing LED illuminations and the excitation light (i.e., not turning them on at the same time), we might be able to monitor intracellular Ca^2+^ dynamics during the period of optogenetic stimulus; we did not do this with constant LED illuminations in this work, because the bright LED output was found to partially leak through the emission filters of the microscope and act as the background noise for Ca^2+^ imaging. In terms of the biocompatibility, our LEDs passivated with an SU8 layer were able to monitor cell activity for *ca.* 1.5 h at room temperature. We expect that this period can be further extended if cells on the array could be kept at ∼37°C by a fixed heating stage. Last but not least, the spatial resolution of our array is currently limited by the omnidirectional emission from LEDs and can be improved by adding light guide or microlens layers in the future.

## Methods

All methods can be found in the accompanying Transparent Methods supplemental file.

## Data and Code Availability

All data generated or analyzed during this study are included in this published article and its Supplemental Information.

## Supplemental Information

Document S1. Transparent Methods and Figures S1–S7
