# Supplementary material for: Single-Cell Optogenetic Control of Calcium Signaling with a High-Density Micro-LED Array
Source: iScience. 2019 Oct 19;21:403–12. doi: 10.1016/j.isci.2019.10.024 (PMC6889635; doi:10.1016/j.isci.2019.10.024)
Supplement: Document S1. Transparent Methods and Figures S1–S7 [file mmc1.pdf]

ISCI, Volume *21*

Supplemental Information

Single-Cell Optogenetic Control of Calcium Signaling with a High-Density Micro-LED Array

Dacheng Mao, Ningwei Li, Zheshun Xiong, Yubing Sun, and Guangyu Xu

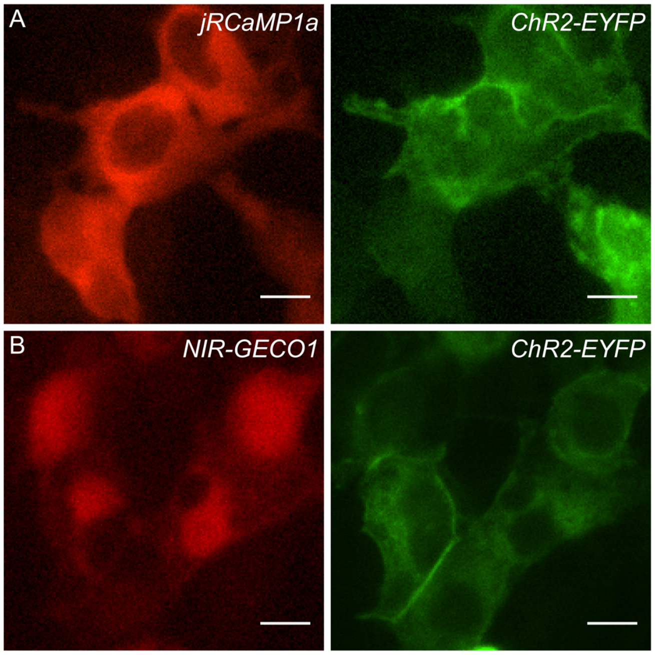

Figure S1. Co-expression of *ChR2* with *jRCaMP1a* or *NIR-GECO1* in HEK 293 cells (related to Figure 1).
Example fluorescence images of *ChR2-jRCaMP1a* (A) and *ChR2-NIR-GECO1* (B) co-expressed cells, pseudo-colored with light red for *jRCaMP1a,* deep red for *NIR-GECO1* and green for *ChR2-EYFP*. Scale bar, 10 μm.

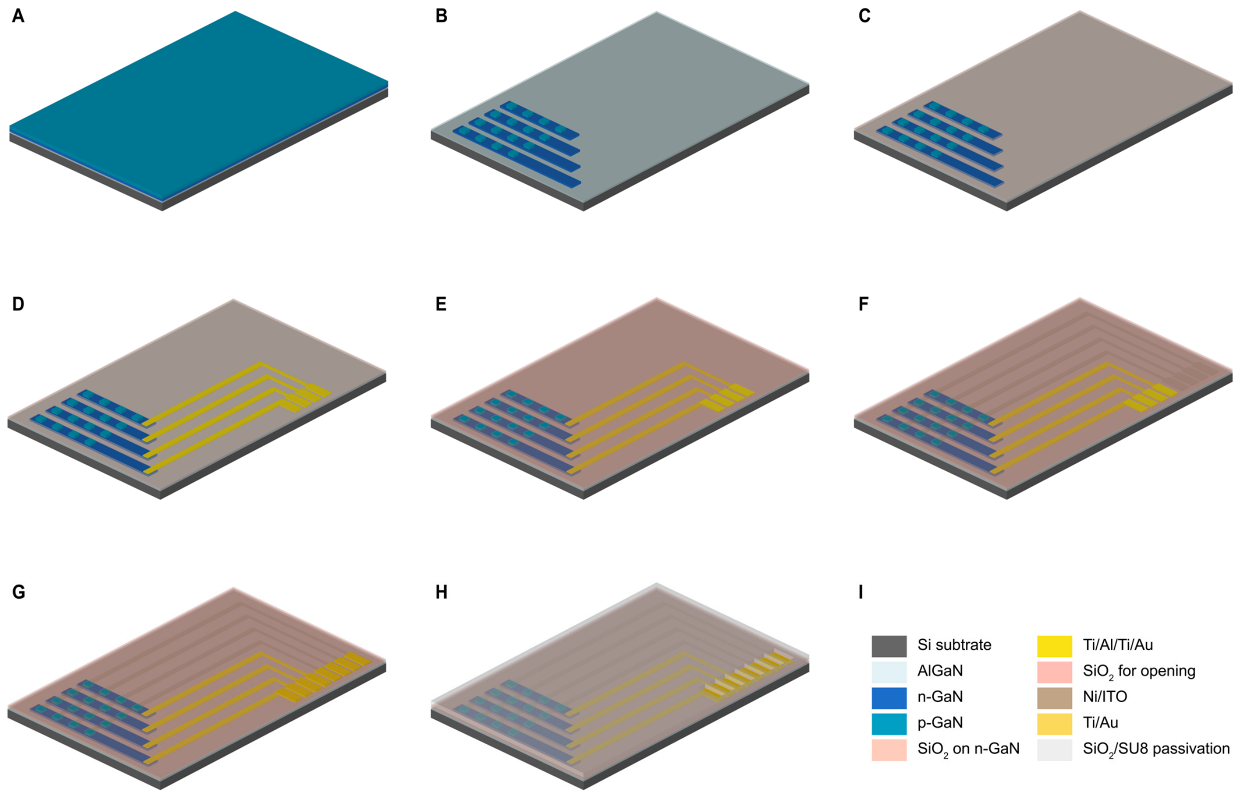

Figure S2. Fabrication flow of the micro-LED array (related to Figure 1).
On an epitaxial GaN-on-Si wafer (A), LED pixels were defined by two RIE steps (B). Four n-GaN islands were passivated by a PECVD based SiO_2 layer (PECVD-SiO_2) (C), followed by evaporating Ti/Al/Ti/Au layers as the n-contact (D). The array was then passivated by a second PECVD-SiO_2 layer, followed by a RIE step to open the pixels and pad areas (E). Next, Ni/ITO (F) and Ti/Au (G) layers were made as p-contact and pad contact, respectively. The entire array was finally encapsulated by PECVD-SiO_2/SU8 layers (H).

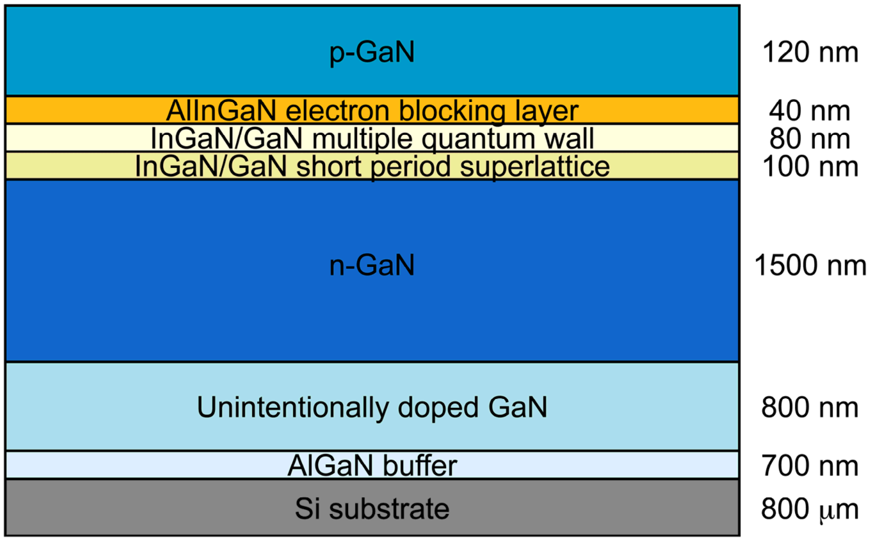

Figure S3. Cross-sectional view of a GaN-on-Si wafer (related to Figure 1).

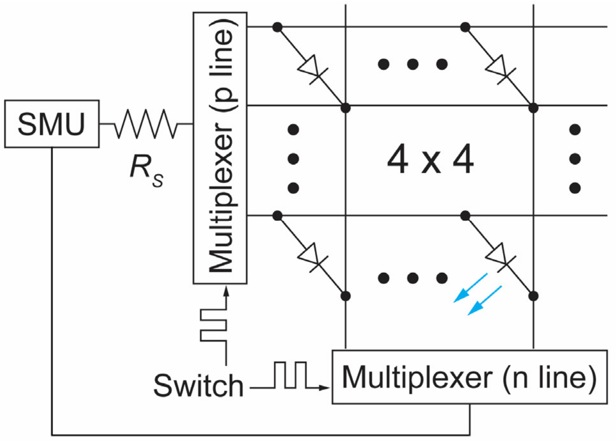

Figure S4. Off-board multiplexing circuit to access individual pixels in a 4-by-4 micro-LED array (SMU: source-measurement unit). R_S represents the applied series resistor (related to Figure 1).

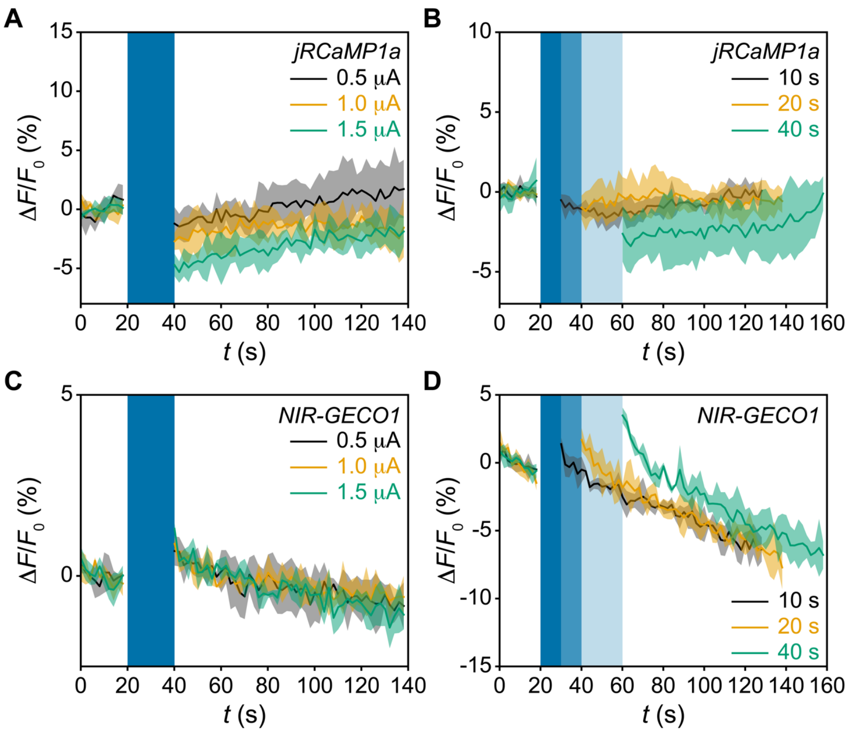

Figure S5. Control cell responses to optogenetic stimulus offered by LEDs (related to Figure 3).

(A) $\Delta F/F_0$ traces from a *jRCaMP1a* control cell with T_{LED} = 20 s and I_{LED} ranging from 0.5 to 1.5 μA. (B) $\Delta F/F_0$ traces from a *jRCaMP1a* control cell with T_{LED} ranging from 10 to 40 s and I_{LED} = 1.5 μA. (C) $\Delta F/F_0$ traces from a *NIR-GECO1* control cell with T_{LED} = 20 s and I_{LED} ranging from 0.5 to 1.5 μA. (D) $\Delta F/F_0$ traces from a *NIR-GECO1* control cell with T_{LED} ranging from 10 to 40 s and I_{LED} = 1.5 μA. Blue windows represent the periods of optogenetic stimulus; solid lines represent the mean value from three consecutive recording periods; shaded areas represent ±1 s.d.

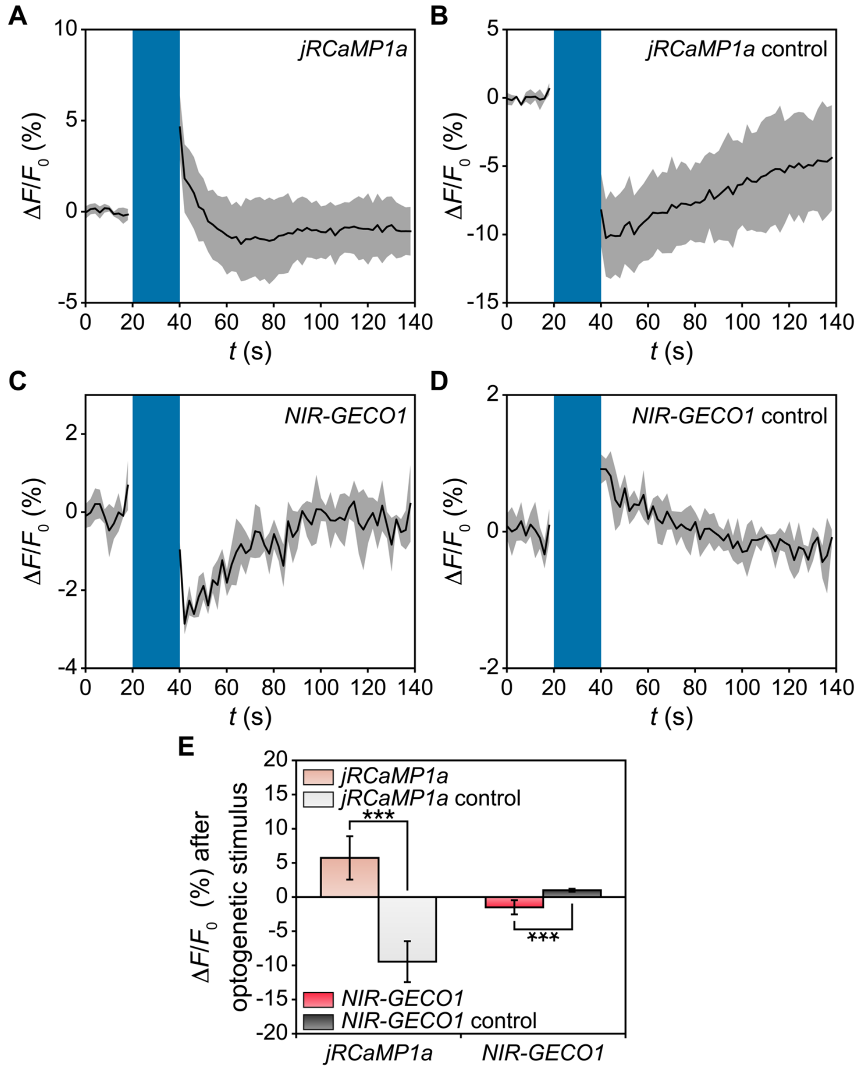

Figure S6. Cell responses to optogenetic stimulus offered by a fluorescence microscope (related to Figure 3).

Representative $\Delta F/F_0$ traces with 20 s microscope-based optogenetic stimulus from a *ChR2-jRCaMP1a* co-expressed cell (A), a *jRCaMP1a* control cell (B), a *ChR2-NIR-GECO1* co-expressed cell (C), and a *NIR-GECO1* control cell (D). In A, B, C, and D, blue windows represent the periods of optogenetic stimulus; solid lines represent the mean value from three consecutive recording periods; shaded areas represent ±1 s.d. (E) Statistical analysis of $\Delta F/F_0$ signals. Error bars represent ±1 s.d. (n = 15 from 5 independent cells in each group, 3 recording periods from each cell); $^{***}P < 0.001$ based on Student's t-test.

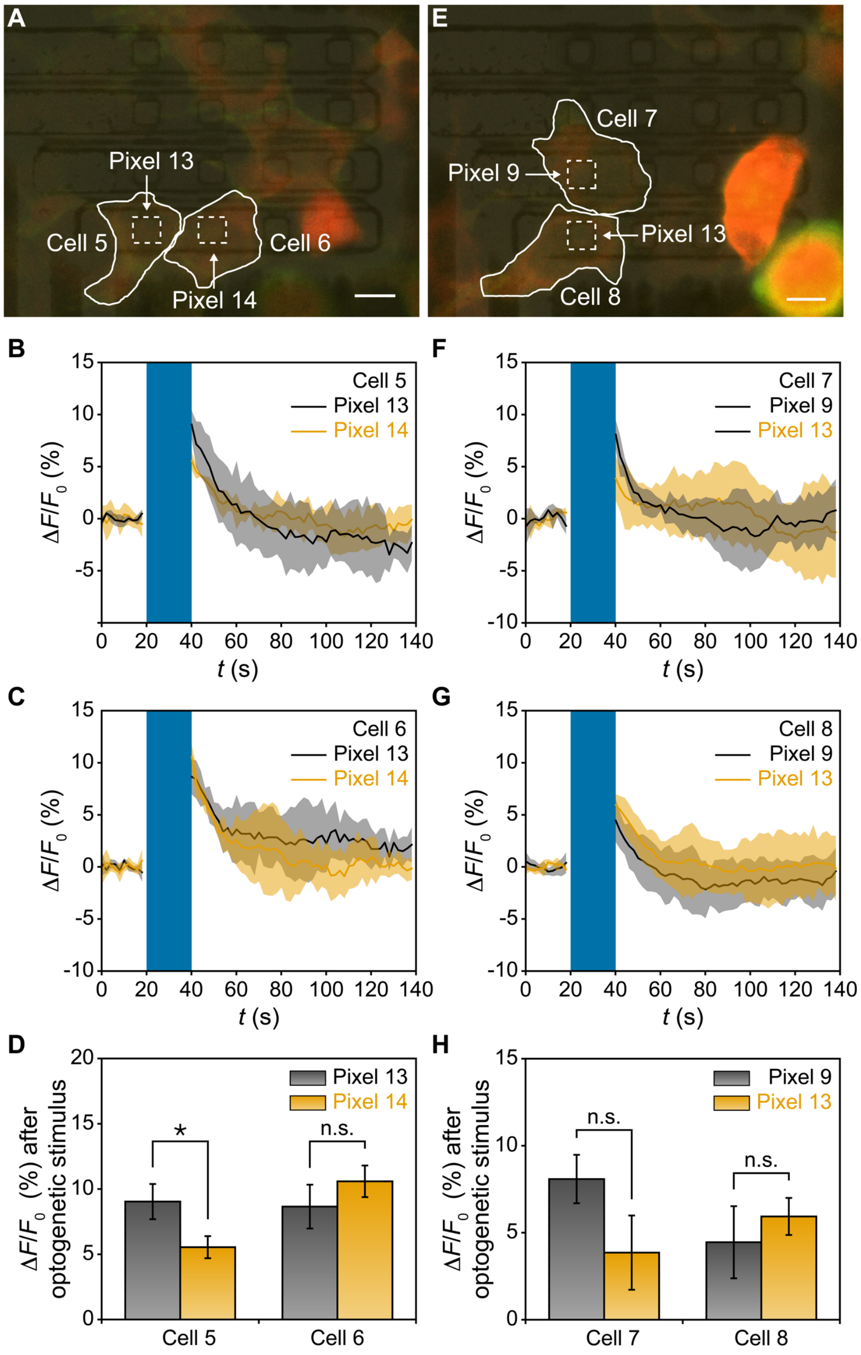

Figure S7. Additional two experiments to evaluate the spatial resolution of the optogenetic stimulus offered by LEDs (related to Figure 4).

(A) One pair of cells (outlined, overlapped with pixels 13 and 14) were sub-1 μm apart. Scale bar, 10 μm. (B) $\Delta F/F_0$ traces from cell 5 that was stimulated by pixels 13 and 14 with T_{LED} = 20 s. (C) $\Delta F/F_0$ traces from cell 6 that was stimulated by pixels 13 and 14 with T_{LED} = 20 s. (D) Statistical analysis of $\Delta F/F_0$ signals from cell 5 and cell 6. (E) Another pair of cells (outlined, overlapped with pixels 9 and 13) were sub-1 μm apart. Scale bar, 10 μm. (F) $\Delta F/F_0$ traces from cell 7 that was stimulated by pixels 9 and 13 with T_{LED} = 20 s and P_{light} ~ 0.71 mW/mm^2. (G) $\Delta F/F_0$ traces from cell 8 that was stimulated by pixels 9 and 13 with T_{LED} = 20 s and P_{light} ~ 0.71 mW/mm^2. (H) Statistical analysis of $\Delta F/F_0$ signals from cell 7 and cell 8. In B, C, F, and G,

blue windows represent the periods of optogenetic stimulus; solid lines represent the mean value from three consecutive recording periods; shaded areas represent ±1 s.d. In D and H, error bars represent ±1 s.d. (n = 3 recording periods); *$P < 0.05$, n.s. $P > 0.05$ based on Student's *t*-test. The I_{LED} values applied to output P_{light} ~ 0.71 mW/mm^2 were 1.6 μA for pixel 9,1.0 μA for pixel 13, and 1.1 μA for pixel 14. These results suggest that our array cannot address individual cells that are sub-1 μm apart.

Transparent Methods

1. Array fabrication.

GaN-on-Si wafers (Enkris Semiconductor) were formed by sequentially growing a AlGaN buffer layer (700 nm), an unintentionally-doped GaN (800 nm) layer, a Si doped n-GaN layer (1500 nm), a InGaN/GaN short period superlattice layer (100 nm), a quantum well layer (80 nm), a AlInGaN electron blocking layer (40 nm), and a Mg-doped p-GaN layer (120 nm) on top of a (111) Si substrate (800 µm). Our LED pixels were defined by two RIE steps. A PECVD-SiO_2 (~200 nm) layer was first applied to passivate n-GaN islands, followed by evaporating Ti/Al/Ti/Au (10/70/10/120 nm) layers as the n-contact. The array was then passivated by a second PECVD-SiO_2 (~200 nm) layer, followed by a RIE step to open the pixel- and pad-areas, as well as a 10-s dip in 50:1 buffered oxide etchant to remove residual SiO_2 (if any). Next, exposed p-GaN areas were treated by a dilute HCl solution (10%) for 30 s to etch unintentionally oxidized, if any, GaN regions, and contacted by sputtered Ni/ITO (5/120 nm) layers. Afterwards, Ti/Au (10/200 nm) layers were deposited to create the pad contact. The entire array was then annealed at 500 ºC for 5 mins in an O_2 atmosphere (20 sccm, 120 mtorr) to reduce the contact resistance. Finally, the array was encapsulated by PECVD-SiO_2 (~200 nm)/SU8 (14 μm) layers before the cell experiment.

2. Cell seeding on PDMS pieces.

PDMS pieces were prepared with a ~200 μm thickness according to the manufacturer's recommendation. Before cell seeding, each PDMS piece was sterilized by 30-minute ultrasonication in 70% ethanol, dried with compressed air, and placed in a 48-well plate (Fisher Scientific). To improve the cell adhesion, we applied a 7-min UV-Ozone treatment (MODEL30, Jelight) to activate the PDMS pieces, coated them with fibronectin (50 µg/ml in DI water, ThermoFisher) for 1 hr at room temperature, and applied 3 times of 1X phosphate buffered saline (ThermoFisher) wash.

After these steps, HEK 293 cells (ATCC CRL-3216) were passaged by 0.25% trypsin (ThermoFisher) and seeded onto the PDMS pieces at a density of 20,000 cells/cm^2. Cells were cultured in 90% Dulbecco's Modified Eagle Medium (high glucose, no glutamine) (ThermoFisher), supplemented by 10% Fetal Bovine Serum (Sigma-Aldrich), 1% penicillin/streptomycin (ThermoFisher), 1% GlutaMAX (100X, ThermoFisher) and 1% Sodium Pyruvate (100 mM, ThermoFisher), and kept in a humidified incubator (ThermoFisher) at 37 ºC with 5% CO_2.

3. Plasmid purification and cell transfection.

The plasmids encoding *ChR2*, *jRCaMP1a*, and *NIR-GECO1* we used are *pcDNA3.1/hChR2(H134R)-EYFP* (Addgene #20940), *pGP-CMV-NES-jRCaMP1a* (Addgene #61562) and *pDuEx2-NIR-GECO1* (Addgene #113680), respectively. Bacteria containing these plasmids were first cultured in LB Agar plates (added with kanamycin or ampicillin) at 37 ºC overnight. Afterwards, single colonies were picked and cultured in LB broth with agitation at 37 ºC overnight to amplify the number of plasmids. Finally, plasmids were purified by a HiSpeed Plasmid Midi Kit (QIAGEN) according to the manufacturer's recommendation.

Cell culture medium was refreshed with addition of 2 µM *all-trans-retinal* (Sigma-Aldrich) 12~24 hours after cell seeding when cell culture reaches 60-70% confluency. Cells were then transfected by adding 40 µL 1 X OPTI-MEM (ThermoFisher) solution mixed with 200 ng of each plasmid (either with single plasmids or one pair of plasmids for co-transfection) and 0.5 µL Lipofectamine LTX reagent (ThermoFisher) to each well of the 48-well plate.

4. Ca^{2+} imaging under optogenetic stimulus.

Ca^{2+} imaging of HEK 293 cells under optogenetic stimulus was performed 24~48 hours after transfection, with an imaging solution containing 80 mM $CaCl_2$, 20 mM glucose, 23 mM NMDG, 5 mM NaCl, 3 mM KCl, 1 mM $MgCl_2$, and 10 mM HEPES (pH 7.3). Cell experiments were conducted using an epifluorescence upright microscope (FN1, Nikon) equipped with a Zyla4.2 plus sCMOS (scientific complementary metal-oxide semiconductor) camera (Andor, USB 3.0) and a SPECTRA X light engine (Lumencor). Cells were

imaged with a CFI6O Fluor 20× water immersion objective lens (NA = 0.5, Nikon) at room temperature. Specifically, we applied: (1) a 6.2 mW 575/25 nm excitation light, a 585 nm long-pass dichroic mirror, and a 632/60 nm emission filter to image *ChR2-jRCaMP1a* co-expressed and *jRCaMP1a* control cells; (2) a 23.1 mW 640/30 nm excitation light, a 660 nm long-pass dichroic mirror, and a 665 nm long pass emission filter to image *ChR2-NIR-GECO1* co-expressed and *NIR-GECO1* control cells; (3) a 3.92 mW 470/24 nm excitation light, a 495 nm long-pass dichroic mirror, and a 520/40 nm emission filter to examine the *ChR2* expression by EYFP imaging. Optogenetic stimulus was offered by either micro-LEDs or a 3.92 mW 470/24 nm light from the SPECTRA X light engine. Fluorescence was sampled at 0.5 frame per second (100 ms exposure time per frame, no binning). The micro-LED array was current biased using a B2902A source-measurement unit (Keysight), which was triggered by FN1 to synchronize with the camera. The microscope-based stimulus was synchronized with the camera using Nikon-Elements Advanced Research software (Nikon).

5. Device characterization.

LED pixels were biased at I_{LED} = 0.1 – 2 μA at a step of 0.1 μA by Keysight B2902A, with a 400 kΩ series resistor (R_{S}) applied to protect the array damage due to improper voltage levels. Accordingly, the V_{LED} values in measured *I-V* curves were corrected by subtracting the voltage drop across R_{S}. During the pulsing test, the select pixel was pulsed at I_{LED} = 2 μA with a 10 ms-duration; I_{light} of the select pixel was recorded by FN1 for one second (1 ms exposure time per frame, no binning or filter cube was applied).

Optical power of micro-LEDs was measured by a digital optical power and energy meter console (PM100D, Thorlabs) connected with a photodiode power sensor (s120C, Thorlabs). The wavelength correction of the power meter was set to 462 nm to match the dominant photoluminescence wavelength of as-made micro-LEDs. Spatial profile of the output light was also captured by FN1 with 1 ms exposure time per frame (no binning or filter cube was applied).

6. Statistical significance.

Statistical analysis in Figs. 3, 4, S6, and S7 was based on Student's *t*-test (two-tailed, independent two-sample *t*-test).
